# Tibet as a Potential Domestication Center of Cultivated Barley of China

**DOI:** 10.1371/journal.pone.0062700

**Published:** 2013-05-03

**Authors:** Xifeng Ren, Eviatar Nevo, Dongfa Sun, Genlou Sun

**Affiliations:** 1 College of Plant Science and Technology, Huazhong Agricultural University, Wuhan, China; 2 Department of Biology, Saint Mary’s University, Halifax, Nova Scotia, Canada; 3 Institute of Evolution, University of Haifa, Mount Carmel, Haifa, Israel; University of Florence, Italy

## Abstract

The importance of wild barley from Qinghai-Tibet Plateau in the origin and domestication of cultivated barley has long been underestimated. Population-based phylogenetic analyses were performed to study the origin and genetic diversity of Chinese domesticated barley, and address the possibility that the Tibetan region in China was an independent center of barley domestication. Wild barley (*Hordeum vulgare* ssp. *spontaneum*) populations from Southwest Asia, Central Asia, and Tibet along with domesticated barley from China were analyzed using two nuclear genes. Our results showed that Tibetan wild barley distinctly diverged from Southwest Asian (Near East) wild barley, that Central Asian wild barley is related to Southwest Asian wild barley, and that Chinese domesticated barley shares the same haplotypes with Tibetan wild barley. Phylogenetic analysis showed a close relationship between Chinese domesticated barley and the Tibetan wild barley, suggesting that Tibetan wild barley was the ancestor of Chinese domesticated barley. Our results favor the polyphyletic origin for cultivated barley.

## Introduction

Barley, a founder crop of old World Neolithic food production, and one of the earliest domesticated crops [1], [2], is one of the main cereals of the Mediterranean belt of agriculture. The spread of crops from their domestication areas involved the dispersal of crop plants well beyond their progenitors’ native range and adaptation to new environments [3]. It has been well demonstrated that cultivated barley (*Hordeum vulgare* ssp. *vulgare*) originated from wild barley (*Hordeum vulgare* ssp. *spontaneum*). The geographic distribution of wild barley in the near East Fertile Crescent was well defined, and the near East Fertile Crescent is considered as the only location where barley was domesticated by some scientists [2], [4]–[8]. However, the discovery of *H. vulgare* ssp. *spontaneum* in sites other than the Fertile Crescent such as Tibet, central Asia, Morocco, Libya, Egypt, Crete, and Ethiopia has challenged the prevalent single origin theory on the origin of barley [9]–[16]. It has been reported that *Hordeum vulgare* ssp. *spontaneum* is distributed in the east-Mediterranean basin and the west Asiatic countries, penetrating into the Aegean region and North Africa to Morocco, and extends eastwards to central Asian areas and Tibet of China [2], [17], [18]. Recent molecular evidence suggested Central Asia, 1,500–3,000 km farther east from the Fertile Crescent [12], and Tibet of China [16] as additional centers of wild barley domestications, and supported multiple origins of cultivated barley. Morrell and Clegg [12], and Saisho and Purugganan [19] suggested that a second domestication occurred east of the Fertile Crescent that contributed to Central and East Asian barleys.

Since the discovery of *Hordeum agriocrithon* Åberg, the close wild relative of barley, and *Hordeum vulgare* ssp. *spontaneum* on the Qinghai-Tibet Plateau in China, the origin of Chinese cultivated barley have received more attention and debate [20]–[26]. Morphological, archaeological cytogenetic and isozyme data have demonstrated that wild barley on the Qinghai-Tibet Plateau was different from the Fertile Crescent wild barley [21], [22], [27]–[29]. Ecologically, the Near East wild barley is adapted to warm and dry climates, while the barley on Qinghai-Tibet Plateau is adapted to cold and dry environments [16]. Recent DArT data supported that Tibet is one of the centers of domestication of cultivated barley [16]. The position of Qinghai-Tibet Plateau wild barley in origin and domestication of cultivated barley has long been underestimated.

The resequencing of gene loci within diverse populations has implications for understanding the origins of barley domestication [3], [12]. Protein content is the most important quality trait in barley. The protein content of barley affects the malting and brewing processes, and the operating efficiency of the brewery and the quality of malt used for making beer [30], [31]. In barley, two protein coding genes, *HvNAM-1* and *HvNAM-2*, have been identified and mapped on chromosomes 6H and 2H, respectively [32], [33]. Allelic variation of the *NAM-1* gene for three species of *Hordeum* representing wild and cultivated barley was analyzed [34]. The expression of the *NAM-1* gene should play a role in grain protein content regulation, and loss of functionality of the NAM-1 gene is related to lower grain protein content in *Hordeum* [34].

HTL (*Hordeum* thioredoxin-like) gene encodes a thioredoxin-like protein. Thioredoxins are universally distributed small-molecular-weight thermostable proteins that have the capacity to catalyze dithiol/disulphide exchange reactions [35], [36]. Thioredoxins play an important role during various aspects of plant life, including enzymatic activation, photosynthesis, photorespiration, reactions associated with the citric acid cycle, lipid metabolism, electron transport ATP synthesis/transformation, membrane transport, translation, protein assembly/folding, nitrogen metabolism, sulfur metabolism, hormone synthesis, and stress responses [37]–[40].

Genomic diversity provides the basis of evolutionary change by natural selection and domestication [8]. Domestication and modern plant breeding practices have narrowed the genetic diversity in cultivated plants [8], [41]. Wild barley provides a rich source of potential genetic variation for barley improvement. Gene sequence variations reflect the genetic and evolutionary history of organisms [42]. This paper characterizes nuclear variation of *Nam-1* and *HTL* genes in wild barley from Southwest Asia, Central Asia, and Tibet of China and cultivated barley from China. Population based analyses and phylogenetic analysis were performed to address if Tibet in China was an independent center of barley domestication, the origin and genetic diversity of Chinese domesticated barley.

## Materials and Methods

### Plant Materials

A total of 103 barley accessions were used in this study including 45 wild barley from Southwest Asia, 18 wild barley from Central Asia, 20 wild barley from Tibet of China, and 20 cultivated barley from China (Table S1). No specific permissions were required for these locations/activities. The materials used in this study were provided by USDA (United States Department of Agriculture) and Huazhong Agricultural University barley germplasm collection. The seeds were planted in pots with sand-peat mixture and maintained in a greenhouse. DNA was extracted from young freeze-dried leaf tissue collected from 5 to 10 plants of each accession using the method of Stein et al. [43].

### DNA Amplification and Sequencing

The single copy nuclear gene *HTL* sequences were amplified by polymerase chain reaction (PCR) using the primers TrxF and TrxR, following the protocols given in Kakeda et al. [44]. Primers for amplification of *Nam-1* gene were designed with the computer program “Primer 3” based on the sequence from *Hordeum vulgare* L. (Genebank accession number DQ869678). The forward and reverse primer sequences are Nam-1HF: 5′-TATCAAGCGCCGTAATTTCC-3′ and Nam-1HR: 5′-ATACTGCCGACGTTTCTGCT -3′, respectively. Amplification of DNA was carried out in 40 µl reaction mixture containing 60 ng template DNA, 0.2 µM of each primer, 1.5 mM MgCl_2_, 0.2 mM of each deoxynucleotide (dATP, dCTP, dGTP, dTTP), 1.5 unit of *Taq* DNA polymerase (Biolabs, New England) and distilled de-ionized water to the final volume. The mixture was amplified using the BioRad iCycler Thermal cycler. PCR condition was as follows: one cycle of 4 min at 95°C, 40 cycles of 1 min at 95°C, 1 min at 52°C, 2 min at 72°C, followed by 8 min at 72°C.

PCR products were purified using the QIAquickTM PCR purification kit (QIAGEN Inc), according to the manufacturer’s instruction, and then sequenced commercially at the Taihe Biotechnology Co Ltd (Beijing, China). To increase quality of the data, both forward and reverse strands were sequenced independently. In order to avoid the error induced by *Taq* DNA polymerase during PCR amplification, each sample was independently amplified three times and sequenced.

### Data Analysis

Multiple sequence alignments were performed using ClustalX [45]. Nucleotide diversity and tests of neutral evolution were performed using the software program DnaSP 4.0 [46] by Tajima’s [47] π, Watterson’s [48] θ and Fu and Li [49]. Phylogenetic analysis was performed with the computer program PAUP* ver. 4 beta 10 [50]. The neighbour-joining (NJ) method [51] using Tajima-Nei distance was used for phylogenetic construction.

## Results

### Genetic Analysis of the *Nam-1* Sequences from Four Populations

**Figure 1:**
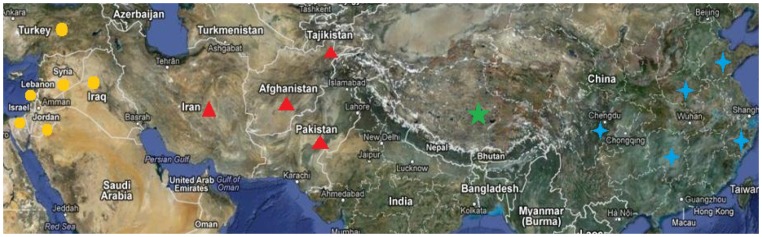
The geographic distribution of sampled four barley nature populations: Southwest Asian wild barley (•), Central Asian wild barley (▴), Tibet an wild barley (★) and Chinese domesticated barley (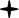).

Sequences of the *Nam-1* were compared from four populations: wild barley of Southwest Asia, Central Asian and Tibet of China, and cultivated barley from China (Fig. 1). The total length of the *Nam-1* sequences amplified was 950 bp. A total of 10 haplotypes were identified in the four natural populations, of which six haplotypes were population-specific, while four haplotypes were shared between populations and only one haplotype was shared by four populations. Comparison of haplotypes among wild barley populations revealed three haplotypes specific to the Tibetan wild barley population and four haplotypes unique to the Southwest Asian wild barley population. However, no haplotype specific to the Central Asian wild barley population was found. Only two haplotypes were identified in the cultivated barley population from China and presented in the Tibetan wild barley population (Table 1).

**Table 1:** Haplotype frequencies of Nam1 gene in four barley natural population.

Nam	Wild barley of Tibet (20)	Landrace barley of China (20)	Wild barley of Center Asia (18)	Wild barley of Southwest Asia (45)
Hap1	0	0	0	0.089 (4)
Hap2	0.50 (10)	0.60 (12)	0.944 (17)	0.467 (21)
Hap3	0	0	0.056 (1)	0.044 (2)
Hap4	0	0	0	0.022 (1)
Hap5	0	0	0	0.267 (12)
Hap6	0	0	0	0.067 (3)
Hap7	0.30 (6)	0.40 (8)	0	0
Hap8	0.10 (2)	0	0	0
Hap9	0.05 (1)	0	0	0.044 (2)
Hap10	0.05 (1)	0	0	0

**Figure 2:**
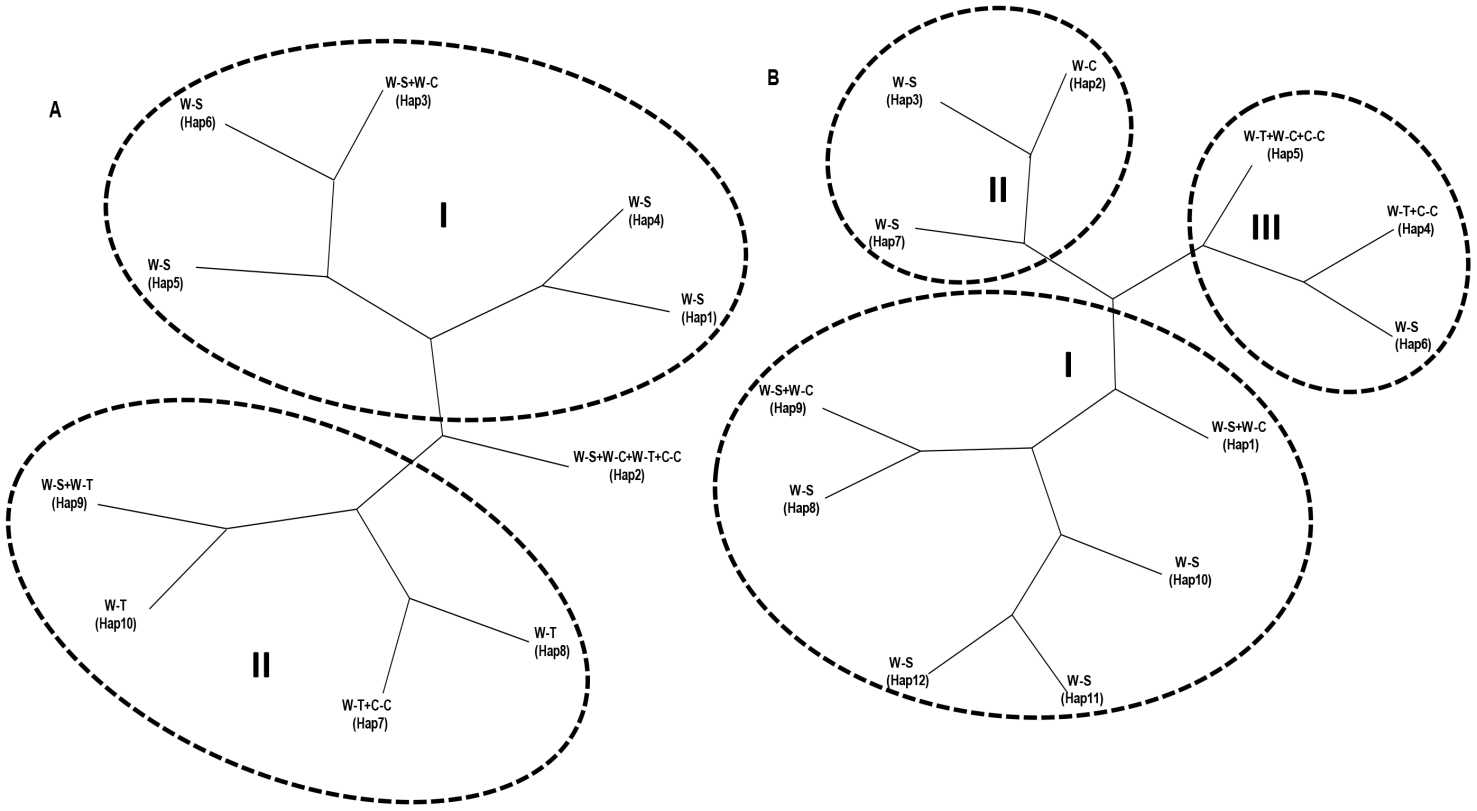
Unrooted phylogenetic trees showing differences between haplotypes and phylogenetic relationships among four groups of wild and cultivated barley accessions for *Nam-1* gene (A) and *HTL* gene (B). W-S (wild barley of Southwest Asia; 45 lines); W-C (wild barley of Central Asian; 18 lines); W-T (wild barley of Tibet; 20 lines); C-C (cultivated barley from China; 20 lines).

The haplotype Hap 2 was the common one, shared among all four populations (Fig. 2A). A total of 5 haplotypes were identified in the Tibetan wild barley population with the majority of accessions (16 of 20) having either haplotype Hap 2 or Hap 7, which are also the two haplotypes present in the cultivated barley population from China. A total of 7 haplotypes were found in the Southwest Asia wild barley population, the majority of accessions were haplotype Hap 2 (46.7%), followed by Hap 5 (26.7%). In the Central Asian wild barley population, we only identified 2 haplotypes, and the majority of accessions (94.4%)) was haplotype Hap 2. The haplotype Hap 7 was unique to barley in China (wild and domesticated barleys); Hap 8 and Hap 10 were unique to the Tibetan wild barley populations, while Hap 1, Hap 4, Hap 5, and Hap 6 were unique to the Southwest Asian wild barley (Table 1).

The genetic diversity and the neutrality test for *Nam-1* gene in different populations are summarized in Table 2. The highest haplotype diversity (Hd = 0.710) and per-site nucleotide diversity (θ = 0.00144±0.00070) were detected in the Southwest Asian wild barley population, followed by the Tibetan wild barley population. The highest nucleotide diversity (π = 0.00101) was detected in the Tibetan wild barley population. Both Tajima and Fu & Li’s neutrality tests were not significant (P>0.05) in all four natural populations. Positive values were obtained for the Tibetan wild barley and Chinese cultivated barley using both the Tajima and Fu & Li tests. In contrast, negative values for both tests were obtained for the Southwestern and Central Asian wild barley except Fu & Li tests for the Southwestern wild barley (Table 2).

**Table 2:** Estimate of Nucleotide Diversity per Base Pair and Test of Neutral for Nam1 gene.

Population	Number ofaccession	Number ofhaplotypes (h)	Haplotypediversity (Hd)	Theta (per site)from S (θ)	Nucleotidediversity (π)	Tajima’sD test	Fu andLi’s D test	Fu andLi’s F test
all	103	10	0.630	0.00162±0.00068	0.00086	−1.13488	0.44682	−0.11216
Wild barley of Tibet	20	5	0.679	0.00089±0.00057	0.00101	0.37128	1.00649	0.95750
Landrace barley of China	20	2	0.505	0.00030±0.00030	0.00053	1.43024	0.64952	0.97941
Wild barley of Central Asia	18	2	0.111	0.00031±0.00031	0.00012	−1.16467	−1.49949	−1.61172
Wild barley of Southwest Asia	45	7	0.710	0.00144±0.00070	0.00096	−0.87418	0.33996	−0.04642

### Genetic Analysis of the *HTL* Sequences from Four Populations

The length of the *HTL* sequences amplified was 935 bp. A total of 12 haplotypes were identified in the four populations. Eight haplotypes were population-specific, while four haplotypes were shared between/among populations. No haplotype was shared by the four populations. A comparison of three populations of wild barley revealed only one each haplotype was unique to the Tibetan wild barley (Hap 4) and the Central Asian (Hap 2) population, while seven haplotypes were unique to the Southwest Asian wild barley. Two haplotypes were identified in the Chinese cultivated barley and were shared with the Tibetan wild barley population (Table 3).

**Table 3:** Haplotype frequencies of HTL gene in population of barley.

HTL	Wild barley of Tibet (20)	Landrace barley of China (20)	Wild barley of Center Asia (18)	Wild barley of Southwest Asia (45)
Hap1	0	0	0.778 (14)	0.20 (9)
Hap2	0	0	0.056 (1)	0
Hap3	0	0	0	0.022 (1)
Hap4	0.10 (2)	0.25 (5)	0	0
Hap5	0.90 (18)	0.75 (15)	0.111 (2)	0
Hap6	0	0	0	0.022 (1)
Hap7	0	0	0	0.133 (6)
Hap8	0	0	0	0.067 (3)
Hap9	0	0	0.056 (1)	0.40 (18)
Hap10	0	0	0	0.022 (1)
Hap11	0	0	0	0.067 (3)
Hap12	0	0	0	0.067 (3)

Three wild barley populations harbored a different high-frequency haplotype. Only two haplotypes were detected in the Tibetan wild barley population with the majority of accessions (18 of 20) having one haplotype (Hap 5). The Chinese cultivated barley population also had only two haplotypes with the majority of accessions (15 of 20) having haplotype Hap 5 that was also detected in two accessions from Central Asia. Nine haplotypes were detected in the Southwest Asian wild barley population, 18 out of 45 accessions had haplotype Hap 9 that was also found in one Central Asian accession. A total of four haplotypes were observed in the Central Asian wild barley population with the majority of accessions (14 of 18) having haplotype Hap 1 that was also presented in 9 out 45 the Southwest Asian accessions (Table 3).

The genetic diversity analysis and the neutrality test results for the *HTL* gene in different populations are summarized in Table 4. The highest number of haplotypes (h = 9), highest nucleotide diversity (π = 0.00157), and greatest haplotype diversity (Hd = 0.785) and per-site nucleotide diversity (θ = 0.00196±0.00086) were observed in the Southwest Asian wild barley population, followed by the Central Asian wild barley population with four haplotypes, nucleotide diversity (π = 0.00075), and per-site nucleotide diversity (θ = 0.00093±0.00060). Nucleotide diversity (π), haplotype diversity (Hd) and per-site nucleotide diversity (θ) in wild barley of Tibet and cultivated barley from China were lower than those in other populations. Tajima and Fu & Li neutrality tests did not significantly depart from neutrality in all four natural populations. Positive values were obtained for Tajima and Fu & Li tests for both cultivated barley of China and wild barley of Tibet. In contrast, negative values were obtained for Tajima and Fu & Li tests for Central Asian wild barley population and Southwest Asian wild barley population (Table 4).

**Table 4:** Estimate of Nucleotide Diversity per Base Pair and Test of Neutral for HTL gene.

Population	Number ofaccession	Number ofhaplotypes (h)	Haplotypediversity (Hd)	Theta (per site)from S (θ)	Nucleotidediversity (π)	Tajima’sD test	Fu andLi’s D test	Fu andLi’s F test
all	103	12	0.797	0.00185±0.00074	0.00151	−0.45468	−0.19209	−0.33579
Wild barley of Tibet	20	2	0.189	0.00030±0.00030	0.00020	−0.59155	0.64952	0.36728
Landrace barley of China	20	2	0.395	0.00030±0.00030	0.00042	0.72261	0.64952	0.76517
Wild barley of Central Asia	18	4	0.399	0.00093±0.00060	0.00075	−0.54951	−0.08478	−0.24097
Wild barley of Southwest Asia	45	9	0.785	0.00196±0.00086	0.00157	−0.54622	−0.08994	−0.27665

### Phylogenetic Analysis

To investigate the relationships among haplotypes, phylogenetic analysis was performed using the neighbor-joining (NJ) method based on Tajima-Nei distance. The phylogenetic tree based on Nam-1 gene showed that the haplotypes detected in the Southwest Asian and Central Asian wild barley formed a cluster (Fig. 2A), and the haplotypes from Tibetan wild barley and Chinese cultivated barley formed a group, except Hap 9 shared between Tibetan and Southwest Asian wild barley (Fig 2A). Phylogenetic analysis based on *HTL* divided the haplotypes into three groups: group I and II with haplotypes from the Southwest Asian and Central Asian wild barley, group III was a mixture of haplotypes from Tibetan wild barley, Chinese cultivated barley, Central Asian wild barley, and Southwest Asian wild barley (Fig. 2B).

**Figure 3:**
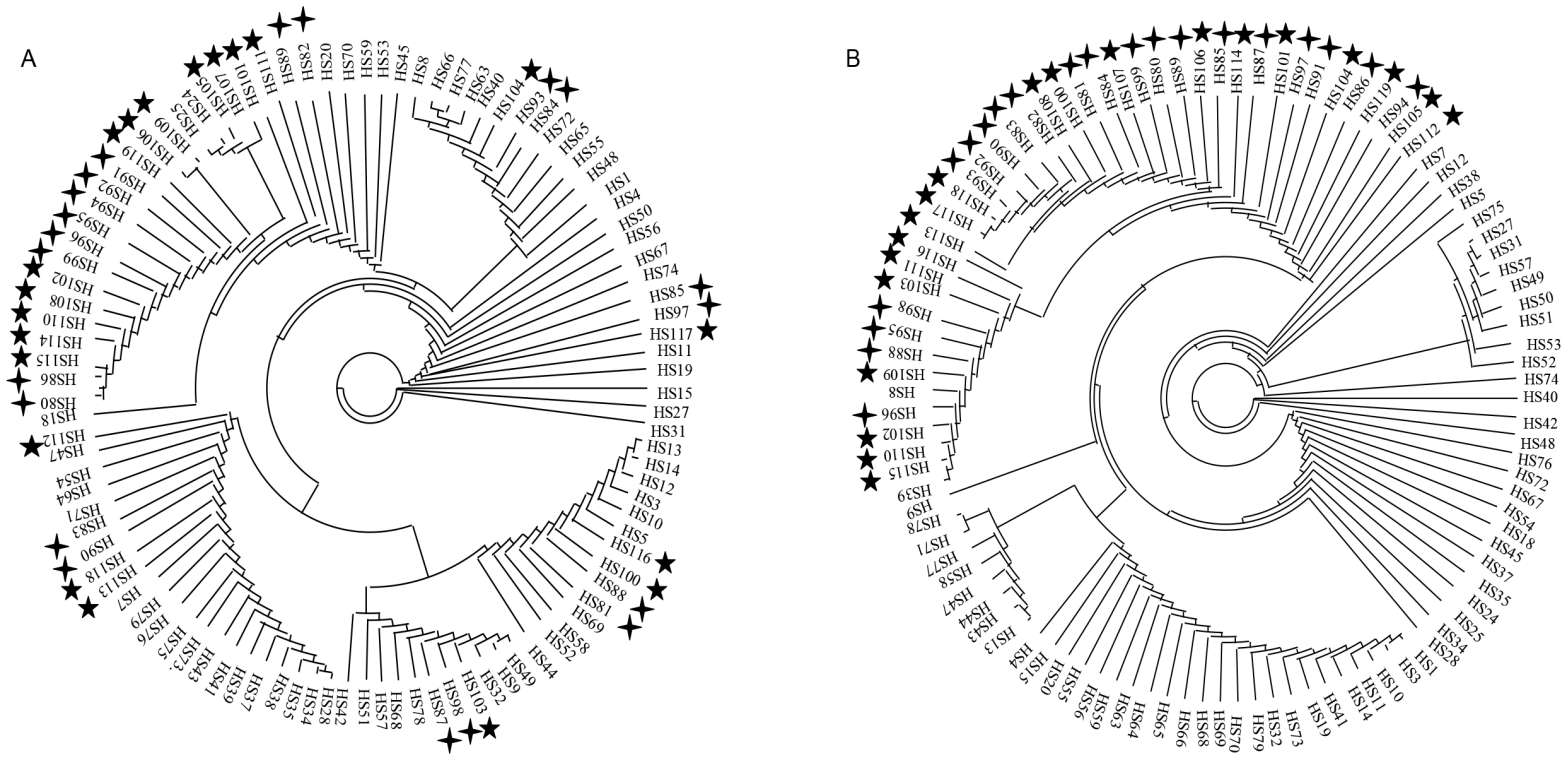
Circle polygenetic showed the phylogenetic relationships among 45 wild barley of Southwest Asia, 18 wild barley of Central Asia, 20 wild barley of Tibet (★ ) and 20 cultivated barley from China (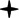) of *Nam-1* gene (A) and *HTL* gene (B).

Phylogenetic analyses were also performed to reveal the relationships among all accessions from the four populations. The *Nam-1* gene tree showed that a majority of accessions of Chinese cultivated barley were grouped together with Tibetan wild barley (Fig. 3A). The phylogenetic tree based on the *HTL* gene clearly grouped all Chinese cultivated barley with all Tibetan wild barley (Fig. 3B).

## Discussion

### Origin of Chinese Domesticated Barley

Two hypotheses were suggested regarding the origin of Chinese cultivated barley. First, China was the original center of the six-rowed barley [52]–[55]. The results from isozyme and ribosomal DNA data supported that Tibetan six-row wild barley was a direct ancestor of six-row and two-row cultivated barley of China [56]. Feng et al. [57] suggested that Chinese cultivated barley might originate from the two-rowed wild barley from Tibet and that the Tibetan six-rowed wild barley was an intermediate form in the process of transformation from two-rowed wild barley to cultivated barley. Analysis of genetic diversity of hordein in wild close relatives of barley from Tibet supported the hypothesis that the Qinghai-Tibet Plateau and its vicinity are the center for the cultivated barley in the Oriental region [58]. The HvGlb1 sequence data favored this hypothesis [59]. Recent DArT data indicated that some Chinese hulless six-rowed barley had been domesticated in the Tibetan Plateau and its vicinity, and suggested that Tibet is one of the centers of domestication of cultivated barley [16], while a second hypothesis suggested the Chinese cultivated barley was introduced from the Near East [60], [61]. AFLP data favored this hypothesis [7]. Sequence polymorphisms and phylogenetic relationships of the *hina* gene in wild barely from Tibet, China suggested that Tibet is unlikely a center of origin for cultivated barley [62]. Our results showed that Chinese domesticated barley shared the same haplotypes with the Tibetan wild barley, and phylogenetic analysis showed a close relationship between Chinese domesticated barley and the Tibetan wild barley (Fig. 3), suggesting that Tibetan wild barley was the ancestor of Chinese domesticated barley. This suggestion was only based on the result from two genes. To further confirm this, we need to analyze more genes sequences.

### Genetic Differentiation between Tibetan wild Barley and the Fertile Crescent Wild Barley

Genetic differentiation between the Oriental and Occidental barley has been reported using allozymes [63]–[65] and rDNA [66]. The total number of alleles and the mean values of genetic diversity of the two-rowed wild barley from Tibet were obviously different from the values of two-rowed wild barley from Israel, Iran, and Turkey [67], [68]. ISSRs and SSRs results indicated that the Tibetan wild barley and the wild barley from the Middle East were distinctly separate from each other [69]. Recent DArT results showed that Tibetan wild barley distinctly diverged from the Near East wild barley [16]. The current results of two nuclear gene sequences showed significant genetic differentiation among wild barley populations. For the *Nam-1* gene, although wild barley populations shared a high-frequency haplotype, large amounts of unique haplotypes were detected in Tibetan and Southwest Asian barley. For the *HTL* gene, the distinct haplotypes were detected in Tibetan wild barley and Southwest Asian wild barley. Phylogenetic analysis also showed a certain degree of separation of haplotypes from Tibetan and Southwest Asian wild barley (Fig. 2). Our results provided evidence to further support that Tibetan wild barley distinctly diverged from the Southwest Asian (Near East) wild barley [16], and revealed that Central Asian wild barley was related to Southwest Asian wild barley. The above-mentioned facts indicated that the Tibetan wild barley is different from the wild barley of the Near East, which indirectly shows that Tibet is an original center and diversity center of cultivated barley. The present data favored the diphyletic origin for cultivated barley [11], [12], [16], [54], [70].

### Loss of Alleles in Domesticated Barley

The domesticated lines harbored fewer haplotypes. Only two haplotypes were found for each gene tested here. Among the 10 haplotypes for *Nam-1* and 12 haplotypes for HTL found in wild barley, only two of them for each gene were present in the domesticated lines, indicating that domesticated lines have lost most alleles of wild types. This is consistent with the analysis of haplotypes of wild and domesticated barley at seven loci –Adh2, Adh3, Amy1, Dhn9, GAPDH, PEPC and Waxy, which in total, 70 different haplotypes in 25 wild barley accessions were observed, while only 17 occurred in the 20 domesticated lines [70]. The per-site nucleotide diversity (θ = 0.00089±0.00057) and nucleotide diversity (π = 0.00101) of *Nam-1* gene in Tibetan wild barley were two-fold of those detected in the Chinese domesticated barley (Table 2). These values indicate a substantial loss of nucleotide diversity in domesticated barley in this gene. Reduction of genetic diversity in the cultivated gene pool of barley has been widely reported [59], [71], [72]. The increase in Tajima’s D from wild (D = 0.37128) to domesticated barley (D = 1.43024, not significant) is also a signature of recent bottleneck [47].

It was notable that the per-site nucleotide diversity of the *HTL* gene in Tibetan wild barley is similar to that detected in the Chinese domesticated barley, while the nucleotide diversity (π) in the Tibetan wild barley was lower than that in the Chinese domesticated barley (Table 4). This might be caused by the natural of this gene. The *HTL* gene encodes a thioredoxin-like protein that plays an important role during various aspects of plant life as mentioned in the Introduction. The Chinese domesticated barley was cultivated in diversified environments, which could cause this functional gene mutation. The positive Tajima’s D value for the Chinese domesticated barley is indicative of over-dominant selection (D = 0.72261), although it was not significant, but accumulated nucleotide diversity in the domesticated barley for this gene.

In summary, our study has provided new insights into barley domestication and the origin of cultivated barley in China. The current results showed that the wild barley of Tibet was different from Southwest Asia. Wild barley of Tibet was the direct ancestor of cultivated barley of China. Wild barley is an important reservoir of genetic diversity and a potential source of beneficial alleles for barley breeding and improvement.

## Supporting Information

Table S1 **The code, accession number, origin and characteristic of 104 barley used in this study.**
(DOC)
