# Supplementary material for: Tibet as a Potential Domestication Center of Cultivated Barley of China
Source: PLoS One. 2013 May 3;8(5):e62700. doi: 10.1371/journal.pone.0062700 (PMC3643926; doi:10.1371/journal.pone.0062700)
Supplement: Table S1 — The code, accession number, origin and characteristic of 104 barley used in this study. (DOC) [file pone.0062700.s001.doc]

Table S1. The code, accession number, origin and characteristic of 104 barley used in this study

| Code | Accession Number | Origon | Characteristic | Code | Accession Number | Origon | Characteristic |
| --- | --- | --- | --- | --- | --- | --- | --- |
| HS1 | PI212305 | Afghanistan | wild, two-row | HS69 | PI662080 | Tajikistan | wild, two-row |
| HS3 | PI219796 | Iraq | wild, two-row | HS70 | PI662109 | Tajikistan | wild, two-row |
| HS4 | PI220664 | Afghanistan | wild, two-row | HS71 | PI662118 | Tajikistan | wild, two-row |
| HS5 | PI227019 | Iran | wild, two-row | HS72 | PI662138 | Turkey | wild, two-row |
| HS7 | PI236386 | Syria | wild, two-row | HS73 | PI662158 | Turkey | wild, two-row |
| HS8 | PI244772 | Pakistan | wild, two-row | HS74 | PI662170 | Turkey | wild, two-row |
| HS9 | PI244774 | Afghanistan | wild, two-row | HS75 | PI662178 | Turkey | wild, two-row |
| HS10 | PI244776 | Afghanistan | wild, two-row | HS76 | PI662188 | Turkey | wild, two-row |
| HS11 | PI244777 | Afghanistan | wild, two-row | HS77 | PI662204 | Turkey | wild, two-row |
| HS12 | PI245739 | Turkey | wild, two-row | HS78 | PI662214 | Turkey | wild, two-row |
| HS13 | PI253933 | Iraq | wild, two-row | HS79 | PI662218 | Turkey | wild, two-row |
| HS14 | PI254894 | Iraq | wild, two-row | HS80 | 042 | Sichuan, China | cultivated, six-row |
| HS15 | PI268243 | Iran | wild, two-row | HS81 | 043 | Sichuan, China | cultivated, six-row |
| HS18 | PI284752 | Israel | wild, two-row | HS82 | 045 | Sichuan, China | cultivated, six-row |
| HS19 | PI293411 | Tajikistan | wild, two-row | HS83 | 046 | Sichuan, China | cultivated, six-row |
| HS20 | PI293412 | Tajikistan | wild, two-row | HS84 | 0177 | Hunan, China | cultivated, six-row |
| HS24 | PI296849 | Israel | wild, two-row | HS85 | 087 | Henan, China | cultivated, six-row |
| HS25 | PI296862 | Israel | wild, two-row | HS86 | 091 | Sichuan, China | cultivated, six-row |
| HS27 | PI296908 | Israel | wild, two-row | HS87 | 0274 | Sichuan, China | cultivated, six-row |
| HS28 | PI354948 | Israel | wild, two-row | HS88 | 0276 | Shandong, China | cultivated, six-row |
| HS31 | PI391100 | Israel | wild, two-row | HS89 | 0384 | Zhejiang, China | cultivated, six-row |
| HS32 | PI401368 | Iran | wild, two-row | HS90 | 056 | Sichuan, China | cultivated, two-row |
| HS34 | PI420911 | Jordan | wild, two-row | HS91 | 0248 | Sichuan, China | cultivated, two-row |
| HS35 | PI420912 | Jordan | wild, two-row | HS92 | 0252 | Sichuan, China | cultivated, two-row |
| HS37 | PI420915 | Jordan | wild, two-row | HS93 | 0279 | Sichuan, China | cultivated, two-row |
| HS38 | PI420916 | Jordan | wild, two-row | HS94 | 0353 | Sichuan, China | cultivated, two-row |
| HS39 | PI420917 | Jordan | wild, two-row | HS95 | 065 | Sichuan, China | cultivated, two-row |
| HS40 | PI466040 | Syria | wild, two-row | HS96 | 0381 | Sichuan, China | cultivated, two-row |
| HS41 | PI466048 | Syria | wild, two-row | HS97 | 0382 | Zhejiang, China | cultivated, two-row |
| HS42 | PI466060 | Syria | wild, two-row | HS98 | 0383 | Zhejiang, China | cultivated, two-row |
| HS43 | PI466086 | Syria | wild, two-row | HS99 | 0385 | Zhejiang, China | cultivated, two-row |
| HS44 | PI466118 | Syria | wild, two-row | HS100 | 01 | Tibet, China | wild, two-row |
| HS45 | PI466130 | Syria | wild, two-row | HS101 | 03 | Tibet, China | wild, two-row |
| HS47 | PI466206 | Syria | wild, two-row | HS102 | 04 | Tibet, China | wild, two-row |
| HS48 | PI466238 | Syria | wild, two-row | HS103 | 013 | Tibet, China | wild, two-row |
| HS49 | PI466249 | Lebanon | wild, two-row | HS104 | 018 | Tibet, China | wild, two-row |
| HS50 | PI466256 | Lebanon | wild, two-row | HS105 | 022 | Tibet, China | wild, two-row |
| HS51 | PI466264 | Lebanon | wild, two-row | HS106 | 023 | Tibet, China | wild, two-row |
| HS52 | PI466296 | Israel | wild, two-row | HS107 | 026 | Tibet, China | wild, two-row |
| HS53 | PI466328 | Israel | wild, two-row | HS108 | 027 | Tibet, China | wild, two-row |
| HS54 | PI466388 | Israel | wild, two-row | HS109 | 028 | Tibet, China | wild, two-row |
| HS55 | PI466498 | Israel | wild, two-row | HS110 | 02 | Tibet, China | wild, six-row |
| HS56 | PI466524 | Israel | wild, two-row | HS111 | 05 | Tibet, China | wild, six-row |
| HS57 | PI466554 | Israel | wild, two-row | HS112 | 06 | Tibet, China | wild, six-row |
| HS58 | PI466586 | Israel | wild, two-row | HS113 | 07 | Tibet, China | wild, six-row |
| HS59 | PI466605 | Iran | wild, two-row | HS114 | 09 | Tibet, China | wild, six-row |
| HS63 | PI554426 | Turkey | wild, two-row | HS115 | 010 | Tibet, China | wild, six-row |
| HS64 | PI466632 | Iran | wild, two-row | HS116 | 011 | Tibet, China | wild, six-row |
| HS65 | PI466699 | Iran | wild, two-row | HS117 | 014 | Tibet, China | wild, six-row |
| HS66 | PI554428 | Turkey | wild, two-row | HS118 | 015 | Tibet, China | wild, six-row |
| HS67 | PI559556 | Turkey | wild, two-row | HS119 | 019 | Tibet, China | wild, six-row |
| HS68 | PI662052 | Tajikistan | wild, two-row |  |  |  |  |
